# Cinema in the training of psychiatry residents: focus on helping relationships

**DOI:** 10.1186/1472-6920-13-90

**Published:** 2013-06-21

**Authors:** Carla Gramaglia, Amalia Jona, Fredrica Imperatori, Eugenio Torre, Patrizia Zeppegno

**Affiliations:** 1 Institute of Psychiatry, Department of Translational Medicine, Università del Piemonte Orientale “Amedeo Avogadro”, Via Solaroli n° 17, Novara, 28100, Italy; 2 Institute of Psychiatry, AOU Maggiore della Carità Hospital, Via Solaroli n° 17, Novara, 28100, Italy

**Keywords:** Education, Empathy, Emotion, Psychiatry trainees, Students, Cinema, Movies, Films

## Abstract

**Background:**

Medical schools are currently charged with a lack of education as far as empathic/relational skills and the meaning of being a health-care provider are concerned, thus leading to increased interest in medical humanities.

**Discussion:**

Medical humanities can offer an insight into human illness and in a broader outlook into human condition, understanding of one self, responsibility. An empathic relation to patients might be fostered by a matching approach to humanities and sciences, which should be considered as subjects of equal relevance, complementary to one another. Recently, movies have been used in medical – especially psychiatric - trainees education, but mainly within the limits of teaching a variety of disorders. A different approach dealing with the use of cinema in the training of psychiatry residents is proposed, based on Jung and Hillman’s considerations about the relation between images and archetypes, archetypal experience and learning.

**Summary:**

Selected full-length movies or clips can offer a priceless opportunity to face with the meaning of being involved in a care-providing, helping profession.

## Background

The century which witnessed the rise of psychology to the ranks of a scientific discipline disregarded the humanistic soul of medicine in the meantime. Despite the widely acknowledged role of clinical empathy as a fundamental determinant of quality in medical care [1] and the growing emphasis on the importance of teaching humanities (e.g. art and literature) to medical students, this approach is often focused on “cultural” issues and to a lesser extent on emotional and relational ones.

Movies have been recently used in medical, and particularly psychiatric education [2-6], through the proposal of movie clubs, cinemeducation lectures [7-11] and/or special modules aimed at teaching about disorders, patient-therapist relationship [2,3,6,12], and in few cases issues such as psychotherapy [13], countertransference [14,15] or psychosocial formulation [16]. Even less popular issues in psychiatry such as stigma and taboo topics like necrophilia have been recently discussed using films [17,18]. Anyway, a symbolic and allegorical approach to movies [19,20] has not received much attention for training purposes so far.

By means of this paper, our aim is to suggest a different perspective on cinema in the training of psychiatry residents, which can be considered a complementary and integrative perspective in addition to its currently described use.

## Discussion

### Theoretical premises: beyond images

Our theoretical background is rooted in Jung [21] and Hillman’s [22] considerations about the relation between images and archetype, and between the archetypal experience (through the work on images) and learning (both from a cognitive and emotional standpoint). Jung described archetypes as archaic, innate images deriving from the collective unconscious. All human beings do share archetypes as inherited potentials. “The archetype is the introspectively recognizable form of *a priori* psychic orderedness” [23]: e.g. the persona, the shadow, the anima, the animus. Archetypes are unconscious and lacking solid content, but these images can acquire “solidity” and eventually turn conscious, through the encounter with empirical facts [23]. Through experience, an archetypical content can become conscious, and actualize its potential in the form of images, behaviors, patterns of interaction with the outside world. Images can therefore play the role of important mediators between conscious learning and unconscious archetypes.

Moreover, our psyche speaks through images [22,24], and psyche can deal with the world or with itself and its functioning. Accordingly, images from a movie, such as those of a dream or a fantasy, can be read at two different levels, an extra-psychical (or objective) one and an intra-psychical one. According to the latter, each character embodies an individual’s part and/or complex. The dream is a theatre and the dreamer is scene, actor, promoter, director, author, public and critic at the same time [25]. Thus the meaning of the movie is far from being univocal: it depends on the message the director wanted to convey; on what the viewer is capable of understanding (the same movie – and this is also true for literature – can be watched and understood differently at different ages, in different moments of one’s life). Finally, it also depends on the Director’s and the viewer’s unconscious, which inevitably emerge in the meaning they assign to the movie.

According to this background, one of the Authors (Eugenio Torre) developed a method based on dynamic images as educational incitements [26]. Dynamic images have an immediate evocative power and are therefore particularly suitable to arouse emotional involvement and to activate unconscious complexes and problems. This approach allows to harmoniously integrate theoretical and technical issues with the experience of working in a group setting. Full-length movies or selected clips can be used and movie images are developed in a group setting. Such setting allows brainstorming and sharing processes; the group members are enriched by the mutual exchange of each member’s perspective and experience. The group members can identify with the different characters and hence experience the situation depicted in the movie from different perspectives. This experience can turn into knowledge, both from a cognitive point of view and, more importantly, an emotional one. The group members can choose and discuss the movie scenes they consider particularly striking, thus raising a reflection on characters. Role-playing of scenes with role inversion is used so as to put feelings, emotions and thoughts into words, which can be elaborated and finally integrated with the help of the group leader.

In accordance with literature concerning cinematherapy [27], movies represent a unique, enjoyable learning instrument [3,28], which can generate a debate [29] and may offer a way to discover what students think about topics which otherwise would remain hidden [17,18,30], or easily overshadowed by technical medical issues [31]. Similar to literature, cinema is not supposed to have a single meaning, and can be considered a complementary way of thinking about the world [32,33]. Moreover, it is through emotion, whose power is to disturb the equilibrium of psyche [34], that screened fiction stirs people’s psyche, nonetheless the emotions aroused by cinema refer to a virtual world and therefore have the potential to increase the individuals’ self-awareness while making them feel relatively “safe” [34,35].

That being stated, movies are chosen according to their potential of bringing to the viewers’ conscious attention themes which can be roughly grouped into three main areas. First, movies can offer the opportunity of mirroring and facing oneself with the meaning of being involved in a helping profession. The multi-faceted relation between the individual and his job includes issues about the individual, the group he works with, its relations and dynamics, the organization he belongs to, motivations, the different methods/approaches adopted by different professionals and, in a broader perspective, vocation and destiny.

Second, some movies are aimed at allowing reflections on one’s shadow sides. According to Jung, everyone carries a shadow, and the less it is embodied in the individual’s conscious life, the blacker and denser it is. Some of these dark, unconscious parts of personality can also show up in one’s job. For example, they can arise as the shadow side of power in the medical profession [36]. Power can lead physicians to swing between an exciting omnipotence (“I can do anything”) and an overwhelming sense of responsibility (“everything depends on me”); such conditions are both dangerous because they neglect the value of relationship and deny the importance of the patients’ role.

Last but not least, the work on movies can be used to discover the feminine (anima) inhabiting every human being and to help developing the feeling function [26]. According to Jung, the anima is the part of individuals enabling them to receive, to hold, to cry; to pay attention to details and shades, i.e. to achieve the discrimination of values depending on the feeling function (considering Jung’s model of the four functions); to go deep into meaning and pain; to promote and take care of life. The work on one’s emotions and feelings is extremely important, and aims both at being aware and at holding them. The emotions aroused by a certain situation allow a deeper understanding of the situation itself. It is the feeling function, not the thinking one, which allows us to manage and recognize values. As Jung stated [37], intellect is undoubtedly useful in its own field, meanwhile it can also generate great confusion when dealing with values. Therefore, the aim of the anima is to mediate between unconscious and conscious (to integrate what is still unknown to conscience) and to give meaning and sense to facts, in order to turn them into experience [22,24].

### Practical procedure

The practical procedure is similar to that of cinematherapy [27], or to the cinema seminars described by Fritz & Poe [28] as part of the residency training program. After watching movies in a thoughtful way, all the issues raised from the trainees’ group are discussed in a psychological context.

The list of movies we commonly use, which is always evolving, includes films that are not specifically targeted to the field of psychiatry, yet chosen for their potential to encourage a discussion on the issues described in the theoretical premises. Issues about (good or bad) helping relationships surround us and can be found, by those willing to, in unexpected places. Just to mention few of them: The Devil’s advocate; An officer and a gentleman; Twelve angry men; To kill a mockingbird; Shadowlands; The remains of the day; The miracle worker; Dead poets’ society; In the bleak midwinter… Therefore, any movie depicting the relationship between two people – a patient and a doctor, a teacher and a student, two friends, two relatives, any two people helping each other - may rise a reflection on relational dynamics and on the characteristics of each person involved in the relationship.

A cinemeducation project involving both psychiatry residents and medicine students is currently ongoing. It started on October 18^th^, 2012, including 12 seminars focused on the doctor-patient relationship. Meetings have been scheduled every fortnight, from 7 p.m. to 10–11 p.m. and about 70 students plus 12 psychiatry trainees are participating. See Table 1 for details about some of the movies seen during the seminars.

**Table 1:** Details about the movies described in the examples

**Title, original title**	**Country, year, directed by**	**Duration**	**The movie plot**
**The seventh floor (Il fischio al naso)**	Italy, 1967, Ugo Tognazzi	108’	Based on a story by Dino Buzzati. A successful businessman, Mr. Inzerna, occasionally develops an unusual physical disturbance: his nose whistles, whenever he breathes. The whistle is cured in a private, luxurious hospital, but new health problems emerge and Mr. Inzerna continues to be cured by the hospital’s staff. In a steady progression, he is first requested, then forced to move from one floor to another. Each floor is less elegant than the lower one, the staff is less appealing and the medical condition of patients is more severe. Inzerna loses his confidence and vitality progressively, and he will eventually die when he reaches the seventh floor.
**The Closet, (Le placard)**	France, 2001, Francis Veber	84’	François Pignon is a dull and colourless man working as an accountant in a rubber factory. When Pignon finds out he is about to be fired, his new neighbour, a retired psychologist, suggests him to spread the rumour that he’s gay, believing that the factory management will no longer fire him if they fear being sued for sexual discrimination. Everybody in Pignon’s life reacts to this news differently, according to their own prejudices and personalities. At first the way people look at Pignon changes, but in the end it is him who really changes and becomes more assertive and manly.
**M**	Germany, 1931, Fritz Lang	117’	Hans Beckert kills the little girls he lures with sweets and toys while whistling a music theme from Grieg’s Peer Gynt. Both the police and the organized crime are on Beckert’s tracks. The latter decides to chase the murderer, with the support of the beggars association, in order to stop the great losses due to the intense search of the police. The criminal organization catches Hans, who is recognized by a blind beggar because of his whistle, and quickly arranges a sort of court to judge him.
**The remains of the day**	UK, USA, 1993, James Ivory	134’	Based on the novel by Kazuo Ishiguro. In post-WWI Britain, Mr Stevens is an inflexible butler whose world made of manners and decorum is challenged by the arrival of Miss Kenton, a housekeeper who eventually falls in love with him.

The seminars described in Table 1 were aimed at arousing a reflection on the meaning of illness, and the reactions to one’s own illness or to a relative/friend’s one. “The seventh floor” was selected for this purpose. It describes Mr. Inzerna’s illness, initially trivialized and underestimated, but eventually leading him to death. At the beginning, Mr. Inzerna is an apparently healthy, active businessman, whose only problem is a weird whistle he makes with his nose when breathing. Viewers can easily identify with such character, and then called to think over the fact that illness is a possibility belonging to every human being (including those involved in helping professions). Moreover, Inzerna’s illness leads him to death, thus encouraging a discussion about the way an illness compels us to face our limits, the greatest and (chronologically) last of which is death itself.

The second issue we wanted to deal with was prejudice, and its impact on relationships and on one’s idea of the world. Being aware of this issue is essential for professionals involved in helping relationships, in order to avoid mistakes due to prejudice and/or excessive identification with those seeking help. We meant to raise a prejudice-challenging debate, and to underscore the importance of keeping an open mind when meeting someone, e.g. a patient. The movies chosen for this issue were “M” and “The closet”. The latter, by means of the character of the retired psychologist, also encourages thinking over the reasons leading to the choice of a helping profession, and on the lights and shadows of this choice.

Last, in order to introduce the work on the concept of anima and the feeling function, we proposed “The remains of the day”. The characters are really suitable for this purpose: Mr. Stevens is an apparently cold, detached man, completely identified with his role and job; he is very inhibited and does not allow his feelings to emerge. On the contrary, Miss Kenton is a warm, sensitive woman, able to use her emotions to acknowledge the value of what happens around her.

Some examples about what was discussed in the groups are shown, according to the issues mentioned above, which we believe may help the reader understand how we work.

### The seventh floor

The main issues raised during the seminar included the lack of empathy and communication between doctor and patient, denying the latter the right to actively take part in the caring process. Students reflected on the different kinds of relationships portrayed in the movie: most of them are somehow cold, detached, objectifying (including doctor-patient relationships, the relationship between the main character and his family); only few (the one with his lover Giovanna, and with the barber) are warm, empathic, characterized by a closeness which is both physical and emotional.

As his clinical condition worsens, the main character (Mr. Inzerna) is moved from the first floor to the next one, up to the seventh floor, where he eventually dies. Each floor corresponds to a more severe degree of illness, and the last two floors host dying people. Nuns and priests only appear on the sixth and seventh floor. From a symbolic point of view, students reflected on the fact that in some cases, when the “technical” issues of medicine fail, “spiritual” (although not in a religious sense) ones must be taken into consideration: listening to patients, to their fears, their need to give a meaning to what is happening to them; to their need of being looked after when they can no longer be cured.

### The closet

The main issues raised during the seminar are represented by prejudice and its influence on relationships. Questions were asked about the relationship between Pignon and his neighbour, a former psychologist who in his youth had been fired because of his homosexuality. Now times have changed and Pignon avoids being fired pretending he is homosexual, as suggested by his neighbour. Confusion in participants emerged about the reasons leading the psychologist to help Pignon. Many students felt he was guided by personal reasons in order to obtain a sort of revenge, a compensation of the injustice he had experienced in his youth. Reflection about this issue was encouraged: everybody, including doctors and psychologists, is certainly influenced by his personal experience. But we then have to distinguish between the two faces of these issues: using one’s job to compensate one’s own deficits or heal one’s wound, and on the other side using one’s experience to help people. The myth of the centaur Chiron has a lot to teach about this: Chiron was a wounded healer, whose wound symbolizes the transformative power of illness and affliction (see also [36]).

### M

The main issues emerged during the seminar were prejudice and the need to go beyond prejudice in order to recognize an individual’s sufferance, even when it is hidden by a monstrous facade. The whole movie leads the viewer to change his perspective about what is traditionally labeled as “good” and “bad”, for example the police and the criminal organization. Challenging certainties is an exercise which every clinician should do in order to keep his mind open in his profession.

The criminal organization succeeds catching the Monster and sets up sort of a “trial”, during which the criminal appointed as “advocate” in defence of Beckert successfully explains a non-judging approach, which is also crucial in the medical profession.

In a more traditional cinemeducation perspective, Beckert’s speech offers an admirable chance to experience the excruciating pain and laceration of a mentally ill person: “I… I can't help myself! I have no control over this, this evil thing inside of me, the fire, the voices, the torment! It's there all the time, driving me out to wander the streets, following me, silently, but I can feel it there. It's me, pursuing myself! I want to escape, to escape from myself! But it's impossible. I can't escape, I have to obey it. I have to run, run… endless streets. I want to escape, to get away! And I'm pursued by ghosts”.

### The remains of the day

Events take place at Darlington Hall, and involve two main characters: Mr. Stevens - Lord Darlington’s butler - and Miss Kenton, his housekeeper. Miss Kenton repeatedly tries to share and communicate her serenity, distress, love, anger, and pain to Mr. Stevens, in the attempt to kindle those emotions he does not seem to feel - or does not want to accept and acknowledge. She suffers and cries for the death of Mr. Stevens’ father in place of Mr. Stevens who is too busy with Lord Darlington’s guests and their aching feet.

Students and trainees were surprised by Mr. Stevens’ apparently cold and detached attitude, and by his inability to receive and greet the warmth brought by Miss Kenton. Moreover, they were struck by Mr. Stevens’ identification with his job and by the importance he gave to it. Therefore, they fluctuated between the identification with either of these two main characters. Miss Kenton represents the feeling function Mr. Stevens denies and rejects, until the end of the movie. Mr. Stevens eventually takes a journey to meet Miss Kenton again – or to meet her for the first time? She is now Mrs. Ben, a wife, a mother and a grandmother, and Mr. Stevens can now feel the emotions aroused by their relationship; he can feel pain, he can suffer. While on one hand this meeting does not allow Mr. Stevens to win back a love which was never acknowledged, on the other hand it allows him to gain a deeper self-awareness.

Students were helped acknowledging this process: at first Mr. Stevens’ anima is projected onto Miss Kenton; only after his (also symbolic) journey he can withdraw this projection, which is necessary to achieve the relation with his own anima, to acknowledge his own emotions and relational resources. This movie allowed trainees to reflect about the feeling function, feminine and anima: “a man without relationships is not a whole because totality can only be reached through the anima, which cannot exist without its counterpart that is always embraced by the You” [21]. The group discussed how, in order to make the unconscious contents accessible, conscience needs to become more feminine, less defined than male conscience, and thus capable to catch what is still hiding in the darkness in a wider perspective [38].

## Summary

Current trends in medical education, increasing theoretical knowledge and focusing on problem solving skills involve the risk of developing a dogmatic approach to medical practice [39]. In typical academic lessons time to reflect on deeper and broader questions about the meaning of being a health-care provider is often lacking; nonetheless physicians – and not only psychiatrists – do find themselves in a relationship with their patients every day, a relationship which is supposed to be helping and empathic. Medical humanities can offer an insight into human illness, but in a broader way into human condition and suffering, perception and understanding of oneself, responsibility to self and others [30].

Our experience with movies showed us that establishing a space/time to talk about these issues is appreciated by trainees and students. They take part in lectures despite the evening timetable; they show enthusiasm in the discussion process elicited by the movie, proving able to move from enjoying a movie to a more reflective and introspective attitude. They really get involved in the discussion, which turns out to be a stimulating and enriching opportunity. Working on the movies mentioned above, for example, allowed the participants to reflect on the importance of empathy, of being in contact with one’s own emotions and feelings so that a real therapist-patient relationship can happen. The debate about prejudice helped understanding how the use of “labels” prevents the possibility to really listen to an individual, and, more specifically, to a patient. Moreover, the character of the retired psychologist in the “The closet” encouraged a discussion about the reasons underlying the choice of a helping profession and the need of being aware of them in order to prevent acting-outs in the therapist-patient relationship.

Our experience strengthens our idea that this kind of work with trainees effectively increases their sensitivity and raises their awareness that they need education in order to improve their relational skills and empathy. Regrettably, we can just report about “impressions”, since a structured assessment of this intervention is still going on. Moreover, the standpoints we discussed reflect our Jungian training and culture. The symbolic approach to a movie is always open to debate, according to the participants’ identity and background.

To consider humanities (particularly literature, visual arts, film and music) and sciences as of equal relevance and complementary to one another might help to bridge the gap between the artistic and scientific faces of psychiatry [28] and to accomplish the goal of relating to patients and their families empathically and compassionately [40].

## Competing interests

The authors declare no competing interests.

## Authors’ contributions

All authors made substantial contributions to conception of the study. CG and PZ drafted the manuscript. All authors read and approved the final manuscript.

## Pre-publication history

The pre-publication history for this paper can be accessed here:

http://www.biomedcentral.com/1472-6920/13/90/prepub
